# Supplementary material for: High Efficiency Mercury Sorption by Dead Biomass of Lysinibacillus sphaericus—New Insights into the Treatment of Contaminated Water
Source: Materials (Basel). 2019 Apr 19;12(8):1296. doi: 10.3390/ma12081296 (PMC6514844; doi:10.3390/ma12081296)
Supplement: Supplementary file 1 [file materials-12-01296-s001.pdf]

materials

Supplementary

# High Efficiency Mercury Sorption by Dead Biomass of *Lysinibacillus sphaericus*. New Insights into the Treatment of Contaminated Water

**J. David Vega-Páez [1], Ricardo E. Rivas [2] and Jenny Dussán-Garzón [1,*]**

1 Microbiological Research Center (CIMIC), Department of Biological Sciences, Universidad de Los Andes, Bogotá 111711, Colombia; jd.vega1754@uniandes.edu.co
2 Department of Chemistry, Universidad de Los Andes, Bogotá 111711, Colombia; re.rivas@uniandes.edu.co
* Correspondence: jdussan@uniandes.edu.co; Tel.: +57-1-3394949

Received: 28 March 2019; Accepted: 12 April 2019; Published: 19 April 2019

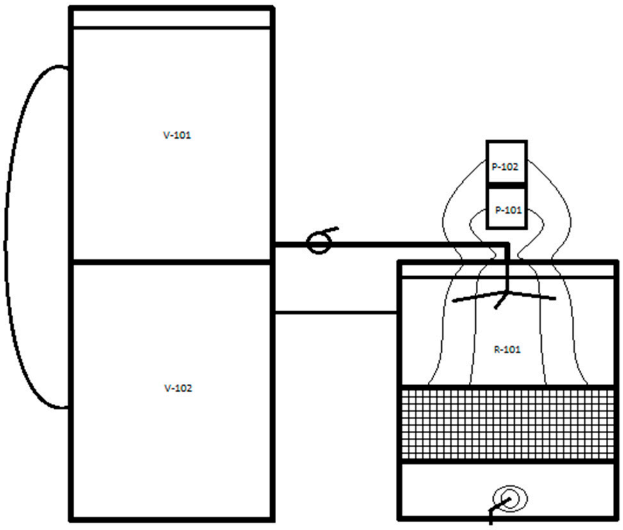


**Figure S1.** Diagram of BFB reactor designed for escalated treatment of Hg. V-101: Hg loading vessel. V-102: Secure vessel. P-101 and P-102: Air pumps for bubbling mixing in RH bed. R-101: Packed reactor with a filter filled with RH.

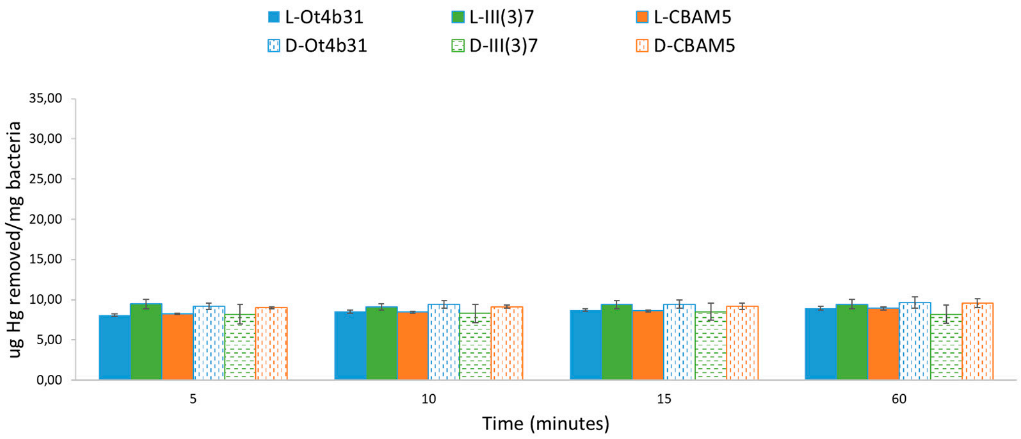


(**a**)

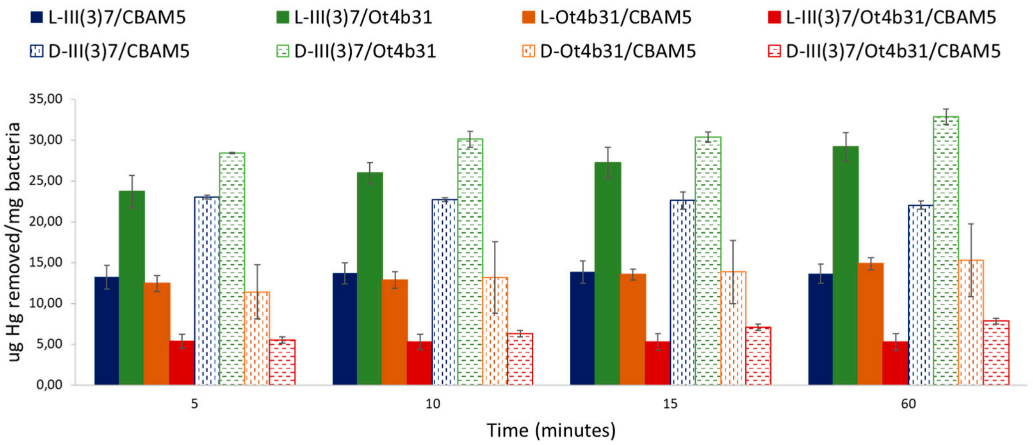


(**b**)

**Figure S2.** Efficiency in mercury removal. (**a**) Single strains efficiency. (**b**) Mixed strains efficiency. (D): Dead. (L): Live.

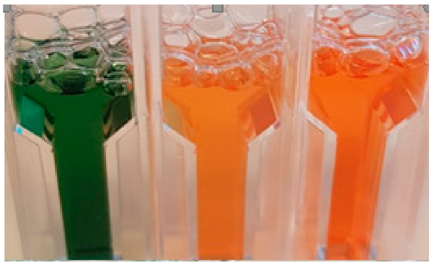

(**a**)

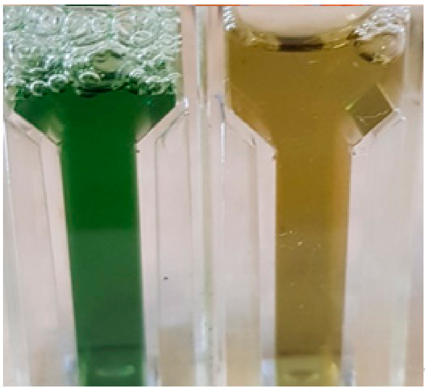

(**b**)

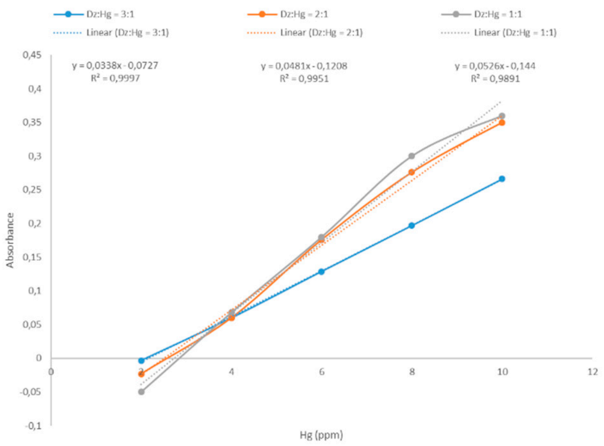


(**c**)

**Figure S3**. Calibration curves for dithizone. (**a**) Reaction in excess of mercury (0 ppm, 8 ppm and 10 ppm). (**b**) Reaction in excess of dithizone (0 ppm, 8 ppm). (**c**) Calibration curves for different proportions of Dz-Hg.

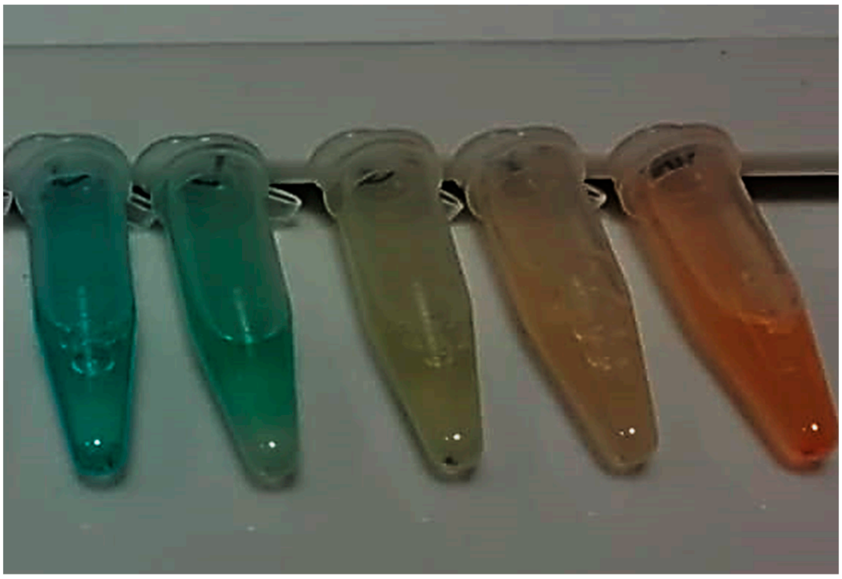

**Figure S4.** Stability of Dz in Triton X114 at different pH. From left to right: pH 3,5,6,7,8.

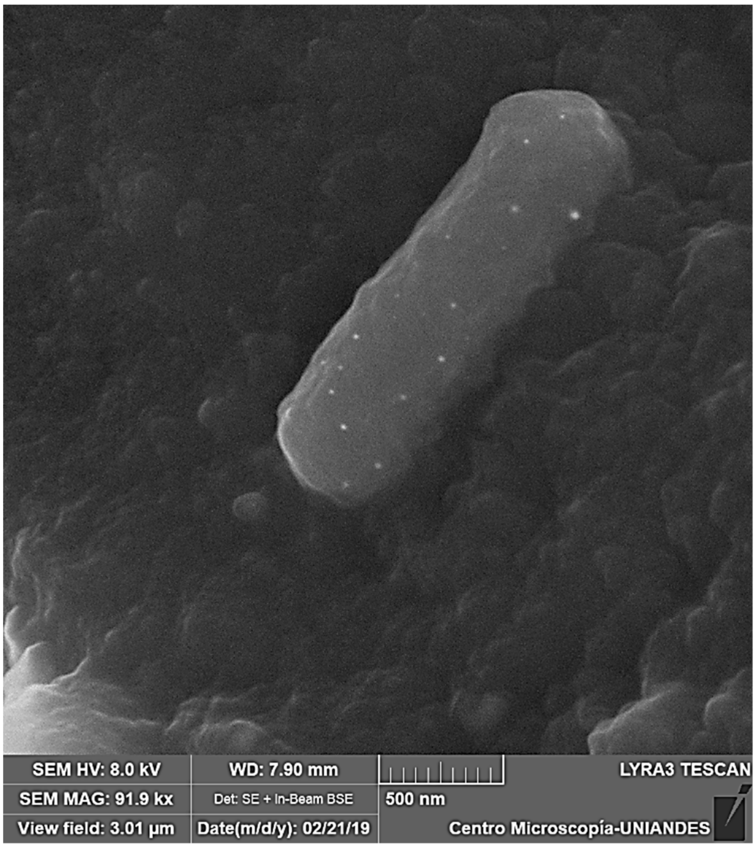


**Figure S5.** Detail of a L. sphaericus cell attached to RH surface.

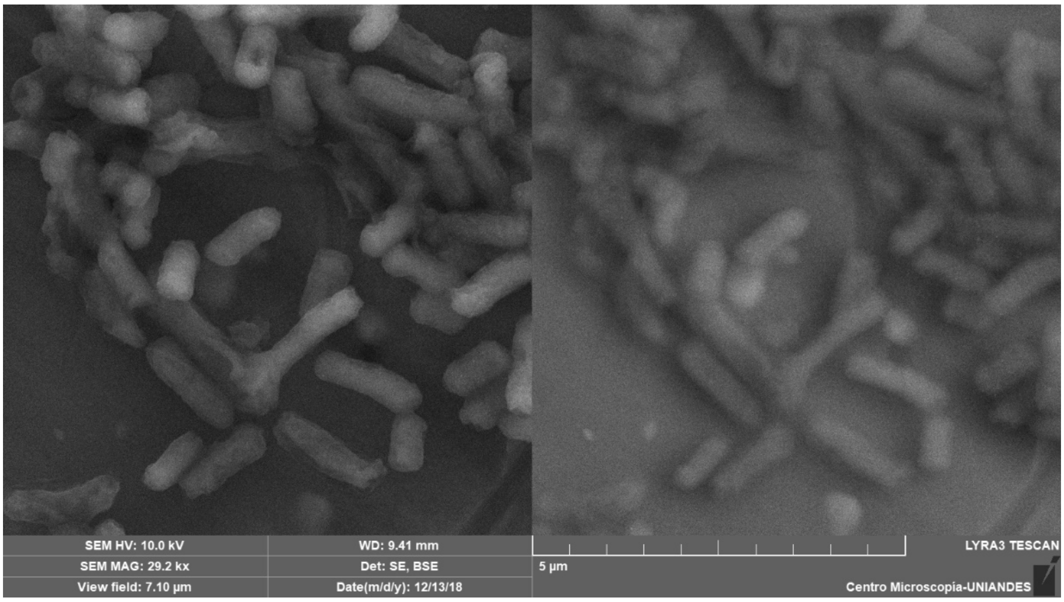


**Figure S6.** EDS-SEM of E. coli K12 C600 after 1 h in contact with $HgCl_2$.

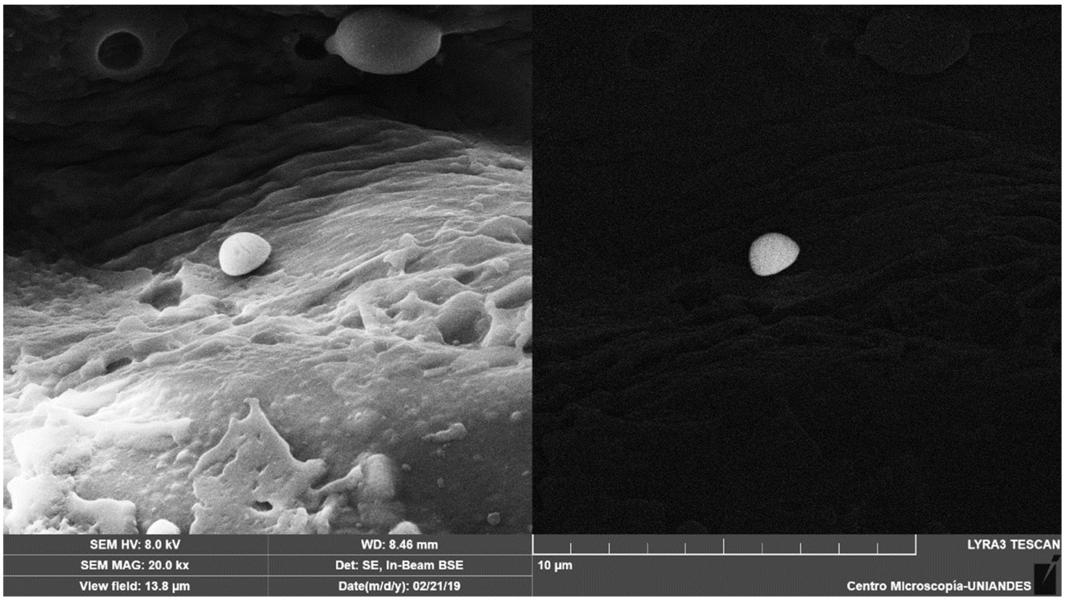


**Figure S7.** RH with a large Hg particle attached to its surface.
